# Overexpression of CYB5R3 and NQO1, two NAD ^+^‐producing enzymes, mimics aspects of caloric restriction

**DOI:** 10.1111/acel.12767

**Published:** 2018-04-28

**Authors:** Alberto Diaz‐Ruiz, Michael Lanasa, Joseph Garcia, Hector Mora, Frances Fan, Alejandro Martin‐Montalvo, Andrea Di Francesco, Miguel Calvo‐Rubio, Andrea Salvador‐Pascual, Miguel A. Aon, Kenneth W. Fishbein, Kevin J. Pearson, Jose Manuel Villalba, Placido Navas, Michel Bernier, Rafael de Cabo

**Affiliations:** ^1^ Translational Gerontology Branch National Institute on Aging National Institutes of Health Baltimore MD USA; ^2^ Nutritional Interventions Group, Precision Nutrition and Aging Institute IMDEA Food Madrid Spain; ^3^ Department of Cell Biology, Physiology and Immunology Agrifood Campus of International Excellence, ceiA3 University of Córdoba Córdoba Spain; ^4^ Department of Physiology Fundación Investigación Hospital Clínico Universitario/INCLIVA University of Valencia Valencia Spain; ^5^ Laboratory of Cardiovascular Science National Institute on Aging National Institutes of Health Baltimore MD USA; ^6^ Laboratory of Clinical Investigation National Institute on Aging National Institutes of Health Baltimore MD USA; ^7^ Graduate Center for Nutritional Sciences Department of Pharmacology and Nutritional Sciences University of Kentucky Lexington KY USA; ^8^ Centro Andaluz de Biologia del Desarrollo, and CIBERER Instituto de Salud Carlos III Universidad Pablo de Olavide‐CSIC Sevilla Spain

**Keywords:** aging, calorie restriction, CYB5R3, metabolic homeostasis, NQO1

## Abstract

Calorie restriction (CR) is one of the most robust means to improve health and survival in model organisms. CR imposes a metabolic program that leads to increased stress resistance and delayed onset of chronic diseases, including cancer. In rodents, CR induces the upregulation of two NADH‐dehydrogenases, namely NAD(P)H:quinone oxidoreductase 1 (*Nqo1*) and cytochrome *b*_5_ reductase 3 (*Cyb5r3*), which provide electrons for energy metabolism. It has been proposed that this upregulation may be responsible for some of the beneficial effects of CR, and defects in their activity are linked to aging and several age‐associated diseases. However, it is unclear whether changes in metabolic homeostasis solely through upregulation of these NADH‐dehydrogenases have a positive impact on health and survival. We generated a mouse that overexpresses both metabolic enzymes leading to phenotypes that resemble aspects of CR including a modest increase in lifespan, greater physical performance, a decrease in chronic inflammation, and, importantly, protection against carcinogenesis, one of the main hallmarks of CR. Furthermore, these animals showed an enhancement of metabolic flexibility and a significant upregulation of the NAD ^+^/sirtuin pathway. The results highlight the importance of these NAD ^+^ producers for the promotion of health and extended lifespan.

## 1 INTRODUCTION

Aging is defined as a progressive decline of intrinsic structural integrity and physiological function. Variations in energy demand, dietary requirements, and cellular energy metabolism contribute to the multiple factors that characterize the aging processes and modulate longevity (Lopez‐Otin, Blasco, Partridge, Serrano & Kroemer, 2013). A reduction in total calorie intake, known as calorie restriction (CR), and various forms of fasting such as intermittent fasting or fasting‐mimicking diets confer a wide range of beneficial effects toward healthy lifespan, with robust protection against cancer (Brandhorst & Longo, 2016; Longo & Panda, 2016). The metabolic fluctuations and the management of cellular bioenergetics induced by fasting cycles are slowly being unraveled and constitute an emerging area of research aimed at developing nutritional interventions to improve health and lifespan.

CYB5R3 and NQO1 are two NADH‐dehydrogenases that play an essential role in the redox control of metabolic homeostasis, a fundamental hallmark of extended longevity (Lopez‐Otin et al., 2013). CR, a robust anti‐aging intervention that extends lifespan in almost all organisms tested, is accompanied by the rescue of age‐associated decline of CYB5R3 and NQO1 function [reviewed in (de Cabo, Siendones, Minor & Navas, 2009; Hyun, Hernandez, Mattson & de Cabo, 2006)]. Likewise, several pharmacological and genetical‐based strategies targeting these enzymes have been employed to ultimately delay aging and age‐related metabolic diseases (Calabrese, Cornelius, Dinkova‐Kostova, Calabrese & Mattson, 2010; Lee et al., 2012; Martin‐Montalvo et al., 2016; Rizvi & Pandey, 2010; Varela‐Lopez, Giampieri, Battino & Quiles, 2016). CYB5R3 catalyzes a two‐step one‐electron transfer reaction in which NADH is oxidized and coenzyme Q (CoQ) is reduced. The elongation and desaturation of fatty acids, cholesterol biosynthesis, and hepatic drug metabolism have all been linked to CYB5R3 (de Cabo et al., 2009). Moreover, CYB5R3 plays a critical role in nitric oxide signaling and vascular function (Rahaman et al., 2015) and in the modulation of lipid metabolism that contributes to healthy lifespan (Martin‐Montalvo et al., 2016). NQO1 catalyzes a two‐electron quinone reduction using NADH or NADPH as cofactor. In addition to its well‐known antioxidant properties [reviewed in (Ross & Siegel, 2017)], NQO1 exerts significant metabolic functions by conferring protection against obesity, hypertension, arterial restenosis, renal injury, and neurodegenerative disorders (Chhetri, King & Gueven, 2017; Hwang et al., 2009; Kim et al., 2011; Son et al., 2015). The C609T (Pro187Ser) null polymorphism of the NQO1 gene is associated with increased risk of complications related to metabolic syndrome, Alzheimer's disease, and an overall increased risk of cancer in humans (Chhetri et al., 2017; Lajin & Alachkar, 2013; Martinez‐Hernandez et al., 2015; Ramprasath et al., 2012). Genetic ablation of NQO1 in rodent models also increases sensitivity to skin carcinogenesis (Iskander et al., 2005) and causes severe dysregulation of lipid and glucose metabolism (Gaikwad, Long, Stringer & Jaiswal, 2001). Recent evidence suggests a role of NQO1 in the modulation of the translational machinery, linking the RNA‐binding function of NQO1 with its metabolic actions (Di Francesco et al., 2016).

In this study, we generated a mouse that is the result of the cross between two strains overexpressing b5‐reductase and NQO1 that are two NADH‐quinone reductases. The offspring that resulted from such mating are referred as “RedTg” mouse. We performed a comprehensive analysis of molecular, physiological, and biochemical outcomes to elucidate the influence of both NADH‐dehydrogenases in the maintenance of whole‐body metabolic homeostasis and to assess whether their upregulation would have a positive impact on health, cancer prevention, and survival. We found that enhanced co‐expression of CYB5R3 and NQO1 mimicked some aspects of CR by conferring health benefits and prolonged lifespan in male RedTg mice. Importantly, RedTg mice survived significantly longer than control mice in a model of induced liver cancer.

## 2 RESULTS

### 2.1 Overexpression of NAD(P)H dehydrogenases extends lifespan and impacts muscle performance

**Figure 1:**
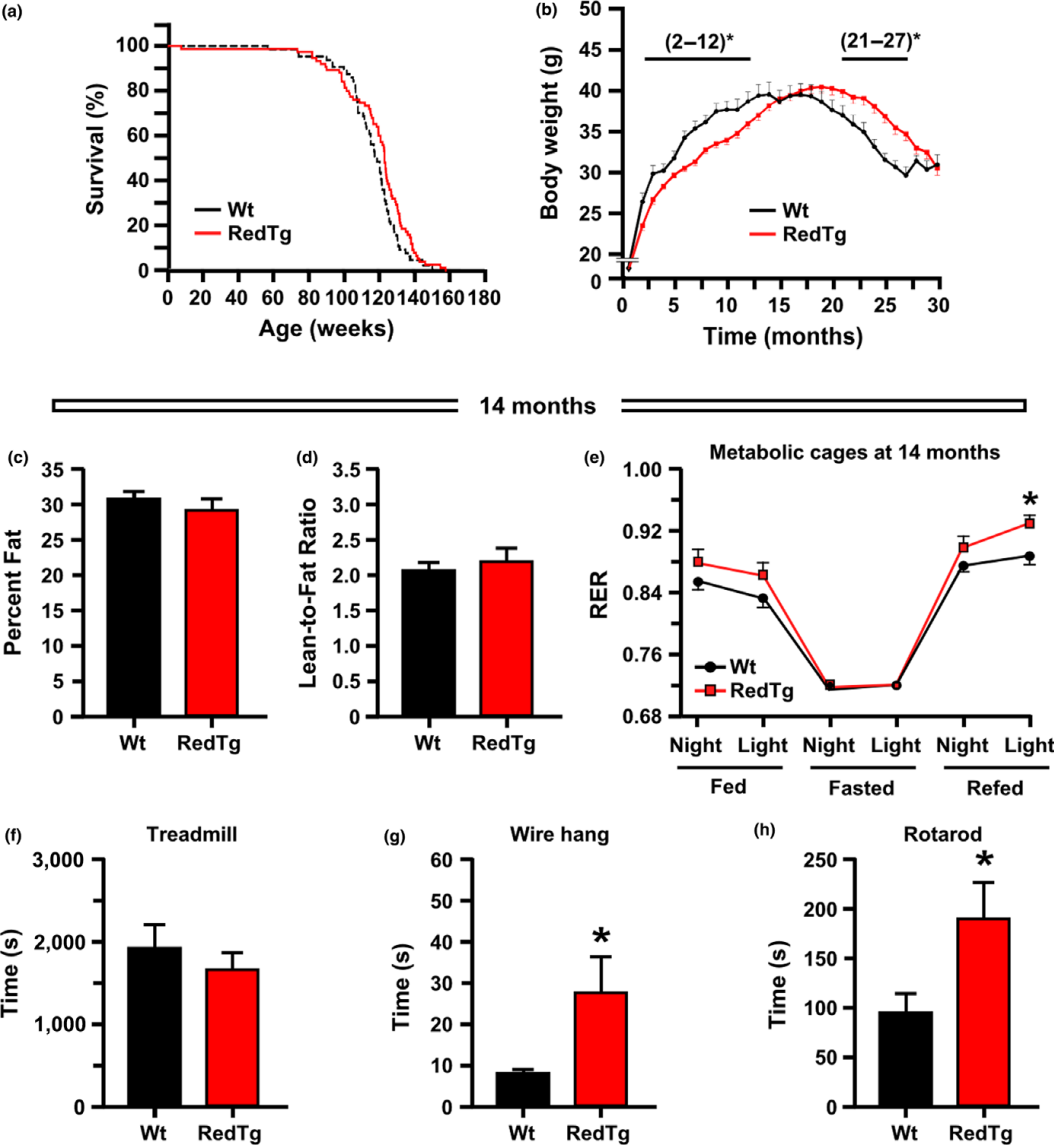
Extension of lifespan and improved health in RedTg mice. (a) Kaplan–Meier survival curve for RedTg (*n* = 75) and Wt (*n* = 64) male mice. Only one Wt animal was censored in the lifespan study. (b) Body weight profile over the lifespan. Bars depict significant differences in body weight between genotypes at the times indicated. Data include all live animals at each time point. (c,d) Effect of CYB5R3 and NQO1 overexpression on body composition. Lean and fat percentages were determined by nuclear magnetic resonance (NMR) in 14‐month‐old mice, *n* = 5–8 mice per group. (e) Fourteen‐month‐old mice were placed into metabolic cages for the measure of respiratory exchange ratio (RER) over a fed‐fasted–refed cycle, *n* = 6–8 per group. (f,g,h) Influence of both NADH‐dehydrogenases on physical performance. Treadmill performance, *n* = 17 mice per group; time to fall from a wire hang, *n* = 5–6 mice per group; time to fall from an accelerating rotarod, *n* = 5–6 mice per group. Data are represented as the mean ± *SEM*. *p <* *.05

A transgenic mouse overexpressing the rat *Nqo1* and *Cyb5r3* genes was created, herein referred to as RedTg. Western blot analysis confirmed the increased levels of NQO1 and CYB5R3 proteins in most tissues tested from RedTg mice compared to their wild‐type (Wt) controls, with the exception of NQO1 in white adipose tissue and CYB5R3 in kidney and liver (Figure S1). A longevity study was carried out (*n* = 64 Wt and *n* = 75 RedTg mice), and the results showed a divergence in the survival curves starting at 110 weeks of age (Figure 1a). The log‐rank test indicated a 4.2% increase in mean lifespan (*p* = .04, χ^2^ = 3.9) and 4.8% extension in 20% survival (*p* = .01, two‐tailed *t* test) in RedTg vs. Wt mice (Figure 1a).

Body weight was monitored across lifespan (Figure 1b). While no difference prior to weaning was found between the two cohorts (6.98 ± 0.18 g vs. 6.58 ± 0.14 g in 16‐day‐old Wt and RedTg mice, respectively), RedTg mice had lower body weight between the age of 2 and 12 months, but were significantly heavier after 21 months of age, as denoted by a right shift of the body weight curve compared with age‐matched Wt controls (Figure 1b; Figure S2a). The switch in body weight trajectory has led us to investigate further the phenotypic changes and potential molecular/metabolic mechanisms that occurred at mid‐life in response to *Nqo1* and *Cyb5r3* overexpression. No differences in body composition [i.e., % body fat, lean mass, or lean‐to‐fat ratio] between genotypes could be detected in 14‐month‐old mice (Figure 1c,d; Figure S2b). We then investigated energy expenditure using an indirect respiration calorimetry system known as the CLAMS metabolic chamber (Martin‐Montalvo et al., 2016). Energy expenditure was calculated from O_2_ consumption and CO_2_ generation. The resultant VCO_2_/VO_2_ ratio, known as respiratory exchange ratio (RER), and heat production were measured over a 91‐hr period. RedTg mice showed a trend toward higher RER during the first (night‐fed, light‐fed), and these differences increased significantly at the third (refed) cycle (Figure 1e), indicating that RedTg mice preferentially used carbohydrates to meet their energy needs as compared to Wt animals. Food consumption was similar in both groups of mice before and after the fasting period (Figure S2c,d), as was heat production and spontaneous ambulatory activity over the course of the fed‐fasted–refed cycle (Figure S2e).

To investigate whether the health improvement and extension of lifespan observed in RedTg mice could be derived from change in insulin sensitivity, we carried out several biochemical tests. Fasting glucose and lactate levels showed no significant differences between the two groups of mice at 14 months of age (Figure S3a,b). Likewise, circulating insulin levels and HOMA‐IR2 index (Figure S3c,d), the oral glucose tolerance test (OGTT) (Figure S3e,f), and the insulin tolerance test (ITT) (Figure S3g,h) showed similar values for RedTg and Wt mice. These results supported the notion that upregulation of NAD(P)H dehydrogenases did not promote changes in peripheral glucose homeostasis in mice of this age‐group.

Cardiovascular performance was then evaluated by treadmill test, while wire hang and rotarod tests were carried out to evaluate the combination of muscle strength with motor coordination and balance. Although both groups of animals exhibited similar treadmill performance (Figure 1f), RedTg mice performed significantly better on the wire hang and rotarod tests (Figure 1g,h), suggesting enhanced overall physical fitness as compared to Wt controls.

**Figure 2:**
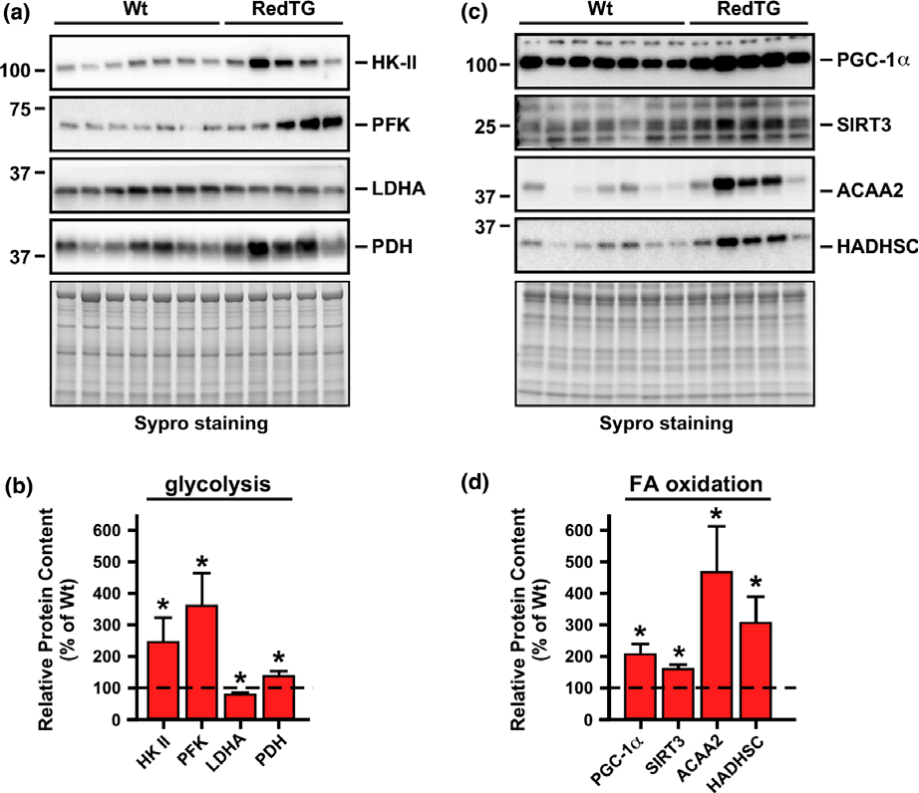
Enhancement of bioenergetics efficiency in muscle in RedTg mice. (a) Total muscle lysates were immunoblotted for key glycolytic enzymes (HK‐II, PFK, LDHA, and PDH). (b) Quantification of protein levels after data normalization with Sypro staining of a duplicate gel. (c) Same lysates were immunoblotted for PGC‐1α, SIRT3, ACAA2, and HADHSC. (d) Quantification of protein levels as in panel B. Dotted line in panels B and D depicts Wt values arbitrarily set at 100%. Data are represented as the mean ± *SEM*,* n* = 5–7 per group. *p <* *.05

Prominent overexpression of both NADH‐dehydrogenases in RedTg muscle (Figure S1) led us to evaluate the abundance of several key metabolic proteins. Western blot analysis of RedTg muscle homogenates showed significantly greater levels of glycolytic markers, such as hexokinase II (HK‐II), phosphofructokinase (PFK), and pyruvate dehydrogenase (PDH), but a decrease in lactate dehydrogenase A (LDHA) content (Figure 2a). Densitometric analysis of these immunoblots is depicted in Figure 2b. Upregulation of these glycolytic markers may reflect higher aerobic glycolytic flux to supply a carbon source for the tricarboxylic acid cycle, a finding consistent with the increase in RER in RedTg mice (Figure 1e). HK‐II and PFK are the main rate‐controlling enzymes of glycolytic flux while PDH activity, which catalyzes the conversion of pyruvate to acetyl‐CoA, modulates mitochondrial glucose and fatty acid fluxes. Mitochondrial fatty acid (FA) β‐oxidation is a metabolic feature promoted by CR and exercise that is controlled by PGC‐1α, a member of a family of transcriptional coactivators, and the protein deacetylase SIRT3 (Hirschey et al., 2010; Liang & Ward, 2006). Western blot analysis showed significant accumulation of PGC‐1α and SIRT3 in muscle of RedTg mice compared to Wt controls (Figure 2c,d). Hydroxyacyl‐coenzyme A dehydrogenase (HADHSC) and acetyl‐CoA acyltransferase 2 (ACAA2) are two mitochondrial enzymes involved in fatty acid β‐oxidation, whose protein levels were substantially higher in RedTg muscles (Figure 2c,d).

Analysis of the activity of mitochondrial complexes from whole muscle homogenates also showed a trend toward upregulation of the activity of mitochondrial complex II, which oxidizes FADH_2_ that is mainly provided by fatty acids (Table S5). Overall, these data suggest enhanced motor function and muscle performance/metabolism in RedTg mice in a CR‐like fashion.

### 2.2 Overexpression of NAD(P)H dehydrogenases enhances glycolysis and impacts lipid metabolism in the liver

**Figure 3:**
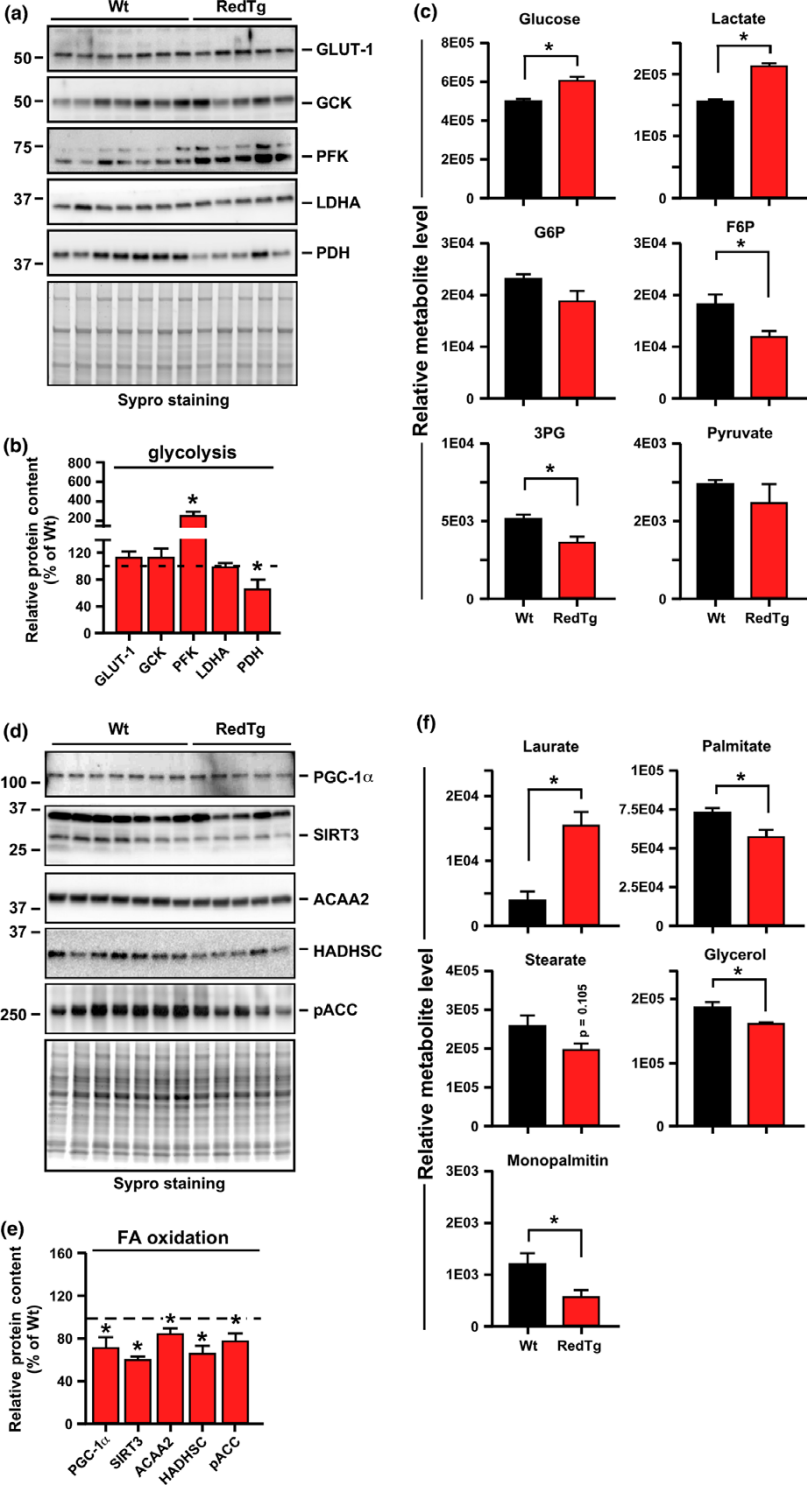
Overexpression of CYB5R3 and NQO1 enhances hepatic glycolysis and impacts hepatic lipid metabolism in RedTg mice. (a) Abundance of key glycolytic enzymes in total liver lysates of 14‐month‐old RedTg mice. Western blotting was carried out using primary antibodies specific for GLUT1, GCK, PFK, LDHA, and PDH,* n* = 5–7 per group. (b) Quantification of protein levels by densitometry. A comparable gel was stained with Sypro (a, *bottom panel*) to confirm equal loading in each lane. (c) Metabolomics analysis of liver extracts was carried out for the determination of glucose, lactate, G6P, F6P, 3PG, and pyruvate. Relative metabolite levels are depicted, *n* = 5–7 per group. (d) Total liver lysates were immunoblotted for PGC‐1α, SIRT3, ACAA2, HADHSC, and phosphorylated ACC. (e) Quantification of protein levels after data normalization with Sypro staining of a duplicate gel. Dotted line depicts Wt values arbitrarily set at 100%. (f) Relative abundance of laureate, palmitate, stearate, glycerol, and monopalmitin from the liver metabolomics analysis. Data are represented as the mean ± *SEM*,* n* = 5–7 per group. *p <* *.05

Western blot analysis of RedTg liver homogenates showed significantly greater PFK protein levels while exhibiting a lower PDH expression than did the Wt liver (Figure 3a,b). However, no differences were observed for glucokinase (GCK), the glucose transporter GLUT‐1, and LDHA, which are involved in the uptake and conversion of glucose (Figure 3a,b). In line with this, metabolomics analysis of RedTg livers showed significant increase in the relative abundance of glucose and lactate, but the intermediates glucose‐6‐phosphate (G‐6‐P), fructose‐6‐phosphate (F‐6‐P), and 3‐phosphoglycerate (3PG) were depleted (Figure 3c). These results combined with the in vivo RER data illustrate a shift in hepatic substrate utilization toward glucose, yielding higher glycolysis and lactate accumulation via reduced expression, and perhaps activity, of the PDH complex (Figure 3a,b).

In RedTg livers, the total expression level of PGC‐1α and SIRT3 as well as HADHSC and ACAA2 proteins was significantly lower compared to Wt livers, consistent with reduced hepatic lipid catabolism (Figure 3d,e). Under these conditions, the pool of the inactive, phosphorylated form of ACC was also diminished in RedTg mice (Figure 3d,e). Metabolomics analysis showed significant depletion in the signals associated with palmitate and stearate, two precursors of polyunsaturated fatty acids, as well as those for glycerol and monopalmitin, two intermediates in the triacylglycerol synthesis (Figure 3f). These results support the notion of increased lipogenesis in RedTg liver. The observed relative increase in laureate would be in agreement with short‐term regulation of hepatic lipogenesis by medium‐chain fatty acids (Geelen, 1994) (Figure 3f).

Analysis of mitochondrial complexes from whole liver homogenates showed no difference in activity between the two groups of mice (Table S5).

### 2.3 Overexpression of NAD(P)H dehydrogenases modulates NAD^+^ metabolism

**Figure 4:**
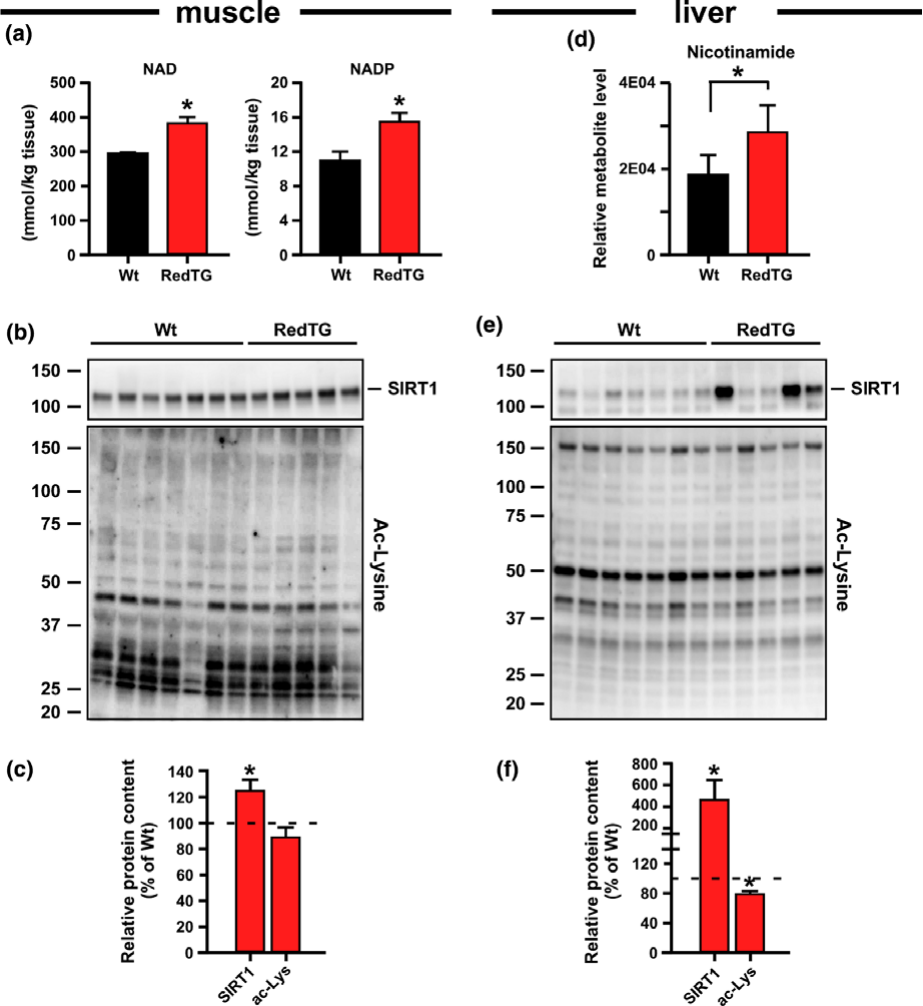
Overexpression of CYB5R3 and NQO1 impacts NAD metabolism. (a) Abundance of NAD and NADP in muscles of RedTg and Wt mice, *n* = 3–4 per group. (b) Total muscle lysates were immunoblotted for SIRT1 and total lysine‐acetylated proteins (Ac‐Lys), *n* = 5–7 per group. (c) Quantification of protein levels after data normalization with Sypro staining. (d) Relative levels of nicotinamide in livers of RedTg and Wt mice from the liver metabolomics analysis. *n* = 5–7 per group. (e) Total liver lysates were immunoblotted for SIRT1 and total lysine‐acetylated proteins (Ac‐Lys), *n* = 5–7 per group. (f) Quantification of protein levels as in panel c. Dotted line in panels c and f depicts Wt values arbitrarily set at 100%. Data are represented as the mean ± *SEM*, *p <* *.05

Sustained glycolysis requires the replenishment of the cellular pool of NAD^+^, a cofactor for several metabolic enzymes, including the class III histone deacetylases known as sirtuins. Because of the essential role of the NAD^+^/sirtuin pathway in the metabolic regulation of aging (Grabowska, Sikora & Bielak‐Zmijewska, 2017), we hypothesized that the overexpression of NQO1 and CYB5R3, two NAD^+^‐producing enzymes, may affect either NAD^+^ metabolism, sirtuin expression and/or activity, or both. As expected, NAD^+^ and NADP^+^ levels were significantly higher in RedTg vs. Wt muscle (Figure 4a), accompanied by an accumulation of SIRT1 protein and a trend toward lower total lysine‐acetylated protein levels in RedTg muscle (Figure 4b,c). Likewise, the buildup of nicotinamide in RedTg livers (Figure 4d) was consistent with active NAD^+^ consumption by sirtuins and the transfer of reducing equivalents important for metabolic use. The RedTg livers had significantly more SIRT1 protein and lower content of total lysine‐acetylated proteins compared to Wt livers (Figure 4e,f).

### 2.4 RedTg mice exhibit protection against inflammation and liver cancer

**Figure 5:**
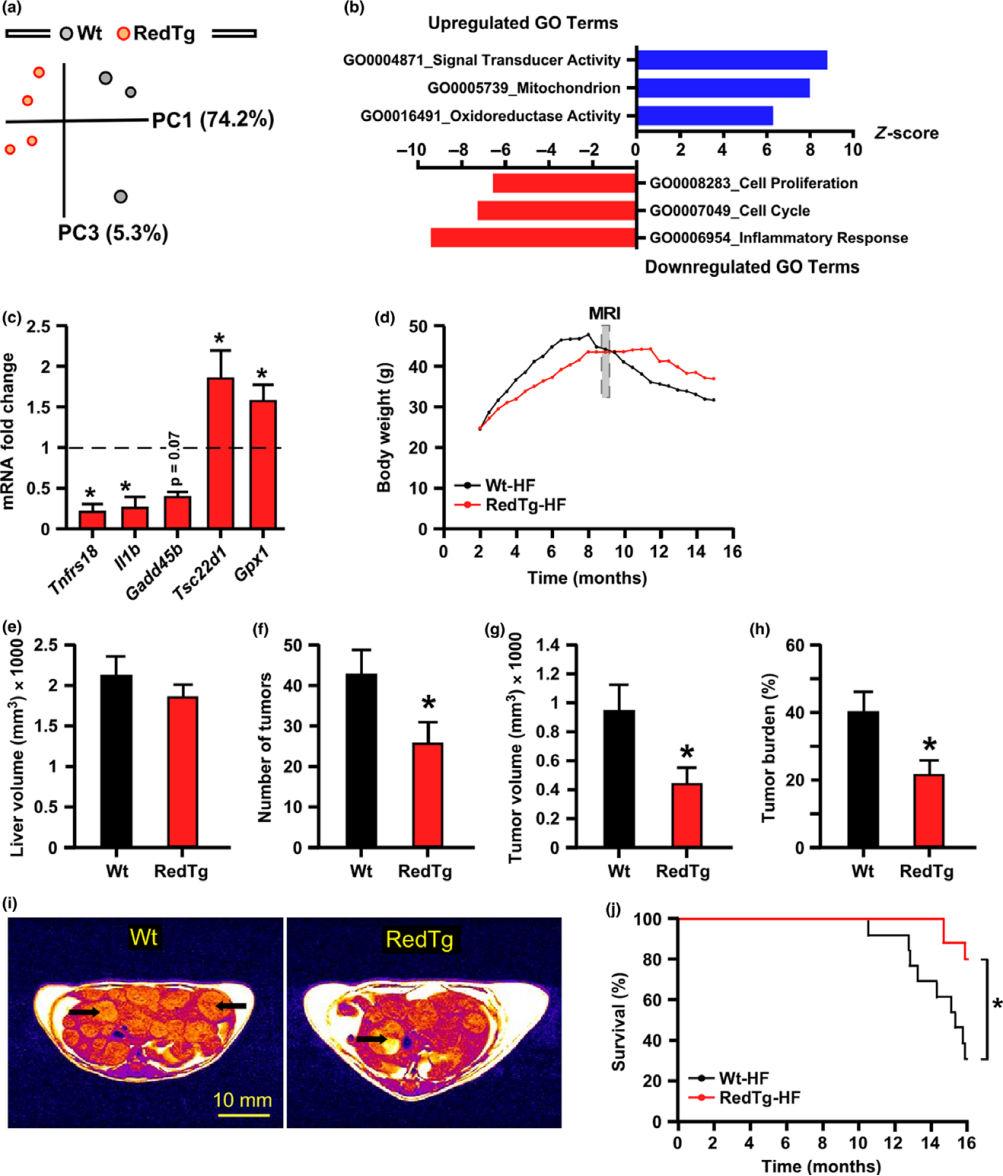
Overexpression of CYB5R3 and NQO1 protects against inflammation and liver cancer. (a) Principal component analysis (PCA) from microarray RNA experiment in livers of 14‐month‐old RedTg and Wt mice; *n* = 3–4 per group. (b) Bars depict gene sets significantly upregulated (blue) and downregulated (red) in RedTg vs. Wt mice. (c) Validation of the microarray data by quantitative RT–PCR analysis. Hepatic mRNA levels of Tnrfs18, Il1b, Gadd45b, Tsc22d1, and Gpx1 in RedTg mice were normalized to Wt controls, *n* = 4–5 per group. (d) Body weight trajectories following treatment of high‐fat (HF) diet‐fed mice with diethylnitrosamine (DEN). Data include all live animals at each time point. MRI analysis was carried out 9 months after starting the DEN protocol for the measure of (e) liver volume, (f) number of liver tumors, (g) tumor volume, and (h) tumor volume ratio, which was calculated as percent of tumor volume divided by liver volume. (i) Representative images of RedTg and Wt livers after 9 months of DEN treatment. Bar, 10 mm. For panels e–h, Wt (*n* = 12), RedTg (*n* = 17). Data are represented as the mean ± *SEM*. *p <* *.05. (j) Kaplan–Meier survival curve for RedTg (*n* = 18) and Wt (*n* = 17) mice includes all animals at each time point until the study termination. The surviving mice were euthanized and considered as censored (RedTg, *n* = 15; Wt, *n* = 8)

Principal component analysis (PCA) was performed to evaluate the liver transcriptome profiles (Figure 5a). Of a total of 497 genes with significantly modified expression, 25.5% (127/497) were upregulated and 74.4% (370/497) downregulated in RedTg vs. Wt livers. The top twenty upregulated and downregulated genes are shown in Tables S6 and S7. These include *Cyp4a12*, a member of the cytochrome P450 superfamily of detoxifying enzymes, and the regulator of cell cycle *Rgcc*. Gene Ontology (GO) term analysis in RedTg livers identified “Inflammatory Response,” “Cell Cycle,” and “Cell Proliferation” among the pathways that were robustly downregulated, whereas “Oxidoreductase Activity” was among the top upregulated pathways (Figure 5b). The mRNA expression level of several transcripts associated with these pathways was measured by quantitative real‐time PCR, which included *Gpx1*, encoding glutathione peroxidase 1, and *Il1b*, a member of the pro‐inflammatory interleukin 1 cytokine family (Figure 5c). Gpx1 mRNA expression levels were elevated while those of Il1b and Tnfrs18, a co‐stimulatory immune checkpoint molecule, were significantly lower by ~90% in RedTg vs. Wt livers (Figure 5c). *Tsc22d1* encodes a leucine zipper transcription factor that plays a role in tumor suppression and *Gadd45b* encodes a protein involved in the regulation of growth and apoptosis. There was significant increase in Tsc22d1 mRNA expression levels and a trend toward lower expression for Gadd45b in response to CYB5R3 and NQO1 overexpression (Figure 5c).

The downregulation of processes involved in liver inflammation and cell cycle progression suggests that the RedTg liver might be protected against cancer development. To test this hypothesis, we treated mice with a single injection of the chemical carcinogen, diethylnitrosamine (DEN), a model of metabolic syndrome‐associated liver cancer (Park et al., 2010). Here, sixteen‐day‐old RedTg and Wt mice were injected with DEN and then put on high‐fat (HF) diet from weaning to 16 months of age. For the first 7 months, the body weight gain in RedTg mice exposed to DEN and HF diet was slower than in Wt mice receiving the same treatment, after which Wt animals experienced a severe drop of body weight while RedTg mice maintained their weight (Figure 5d). MRI scans were acquired at the age of 9 months and revealed that while the liver volume remained comparable between genotypes (RedTg, 1848.2 ± 129.1 mm^3^; Wt, 2115.6 ± 220.2 mm^3^; *p* = .27) (Figure 5e), the number and volume of liver tumors were significantly lower in RedTg vs. Wt mice: [25.8 ± 4.8 vs. 42.7 ± 5.7; *p* = .03] and [449.8 ± 105.4 mm^3^ vs. 942.8 ± 177.5 mm^3^; *p* = .01], respectively (Figure 5f,g). As a result, the tumor burden, defined as the percentage of tumor volume compared to total liver volume, was found to be lower in DEN‐treated RedTg vs. Wt mice [21.9 ± 4.1% vs. 40.6 ± 5.6%; *p* = .01] (Figure 5h,i). Notably, when exposed to DEN and HF diet, the Wt mice exhibited significantly lower longevity than RedTg mice (Figure 5j).

## 3 DISCUSSION

CR without malnutrition reduces the incidence of age‐associated disease and extends lifespan through mechanisms that include improvement of insulin sensitivity and cardiometabolic benefits, accompanied by enhanced cognitive and motor function. Skeletal muscle adaptations to CR and exercise lead to enhanced capacity for glucose utilization and FA oxidation (Navas‐Enamorado, Bernier, Brea‐Calvo & de Cabo, 2017). Despite the lack of effects on peripheral glucose homeostasis, bioenergetic efficiency in skeletal muscle was improved in response to overexpression of CYB5R3 and NQO1, mirroring a CR‐like metabolic profile that was accompanied by enhanced muscle strength, motor coordination, and balance. Metabolic flexibility during CR is characterized by an enhanced capacity to cycle from whole‐body FA metabolism to carbohydrate utilization immediately after feeding (Bruss, Khambatta, Ruby, Aggarwal & Hellerstein, 2010). Analysis of indirect respiration calorimetry data showed significantly greater glycolytic metabolism in RedTg mice. Metabolic reprogramming toward a preferential use of carbohydrates was further confirmed by upregulation of glycolysis in muscle and liver. This metabolic shift in substrate utilization was coupled with a strengthening in redox control and tissue NAD^+^ homeostasis, as evidenced by greater accumulation of NAD^+^ (in muscle) and nicotinamide (in liver) in RedTg vs. Wt mice. Endogenous formation of nicotinamide can occur directly by cleavage of NAD^+^ by NAD^+^‐consuming enzymes such as sirtuins (SIRTs), CD38, and PARPs (Canto, Menzies & Auwerx, 2015; Verdin, 2015). The significant accumulation of SIRT1 protein in muscle and liver extracts of RedTg mice was accompanied by weaker global protein acetylation signal, possibly because of higher SIRT1 activity as compared to Wt mice. Many lines of evidence indicate that sirtuins mediate the effects of CR, and small molecule SIRT1 activators confer broad health benefits likely through SIRT1‐mediated deacetylation of target proteins [reviewed in Ref. Guarente, 2013]. Overall, our findings are in agreement with the role of CYB5R3 and NQO1 as efficient intracellular generators of NAD^+^ for utilization by sirtuins (Ross & Siegel, 2017; Shen et al., 2017), and are consistent with the notion that modulation of SIRT1 and NAD^+^ levels represents a promising approach for the treatment of age‐associated metabolic diseases (Herranz et al., 2010; Imai, 2010; Verdin, 2015).

It is worth mentioning that, after an initial period of weight loss, CR mice reestablish a state of energy balance in which fat mass is preserved or even increased to adjust FA oxidation with its intake plus synthesis (Bruss et al., 2010). Herein, the preference of RedTg mice for carbohydrate utilization was likely associated with higher rate of hepatic lipogenesis—using the ratio of phospho/total form of ACC as surrogate marker—which was further supported by the metabolomics analysis. Conversely, there was clear reduction in key regulators of the lipid oxidation process, including PGC‐1α, SIRT3, ACAA2, and HADHSC in RedTg livers. It has been previously reported that ad libitum‐fed animals on diets that are high in carbohydrates, but low in proteins, have greater adiposity and the longest lifespan (Solon‐Biet et al., 2014), although the association between the intake of protein/carbohydrates and adiposity is still a matter of debate (Fontana et al., 2016; Maida et al., 2016; Solon‐Biet et al., 2015) including prospective cohort studies of humans (Berryman, Agarwal, Lieberman, Fulgoni & Pasiakos, 2016; Pimpin, Jebb, Johnson, Wardle & Ambrosini, 2016). Nonetheless, the maintenance of body weight in older RedTg mice may be due to higher fat accumulation than in Wt controls, in agreement with our recent study that showed a positive correlation between mouse longevity and conservation of body fat (Mitchell et al., 2016). Although lipidomics analysis was not performed in the current study, health improvement and extension of lifespan have been linked to fatty acid chain elongation and desaturation both in flies and mice overexpressing CYB5R3 (Martin‐Montalvo et al., 2016).

Reduction in low‐grade chronic inflammation and anticancer protection are among the CR‐mediated physiological hallmarks that lead to successful aging. Here, the tumor‐promoting effects of DEN were significantly abrogated in RedTg mice by delaying the formation of liver cancer. The development and progression of liver cancer as well as the relative risk of cancer‐related deaths have been associated with chronic systemic inflammation(De Pergola & Silvestris, 2013). Interestingly, ablation of TNF signaling in mice by genetic deletion of the type 1 TNF receptor (*Tnfr1* ^−/−^) markedly impedes the development of obesity‐dependent liver cancer (Park et al., 2010). In our study, overexpression of CYB5R3 and NQO1 resulted in lower hepatic inflammatory signature as well as a lower level of Tnrfs18 mRNA, which encodes a member of the TNF receptor superfamily. Likewise, pathways populated with genes associated with cell proliferation and cell cycle were significantly downregulated in RedTg livers, a finding that was independently validated by the analysis of Gadd45b mRNA expression. Interestingly, reports have shown the existence of cross talk between the TNFα‐NFkB signaling pathway and the coactivator Gadd45β (Kodama & Negishi, 2011; Tian et al., 2011), and strong staining of GADD45β has also been detected in multiple human cancer tissues (Hoffman & Liebermann, 2013). Reciprocal regulation of the putative tumor suppressor gene *Tsc22d1*, which leads to the suppression of *Gadd45b* and that of other cancer‐associated target genes (Iida, Anna, Gaskin, Walker & Devereux, 2007), may also account for the lower rate of tumor progression in RedTg livers.

**Figure 6:**
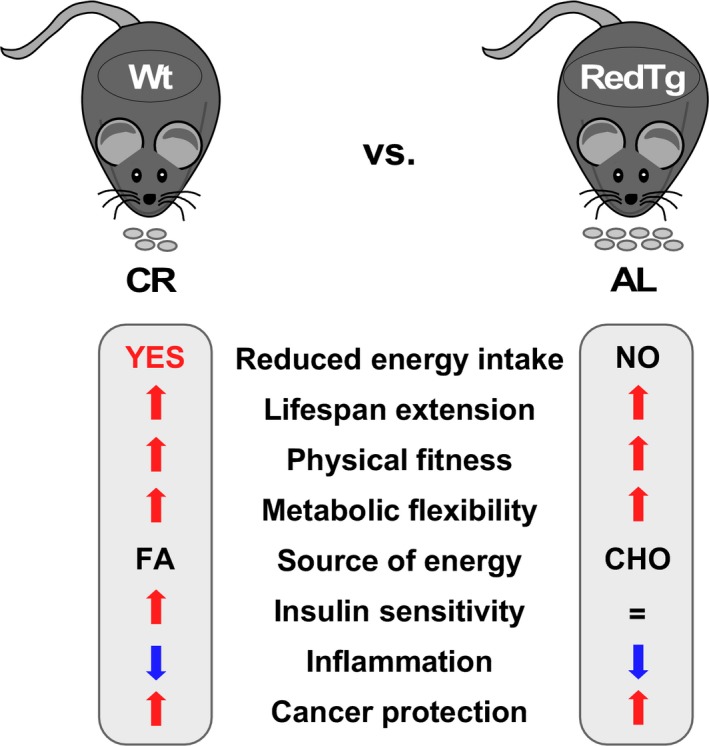
Schematic representation of the main features induced by CR and shared by RedTg mice overexpressing CYB5R3 and NQO1 proteins. Please note that whole‐body physiology was characterized in 14‐month‐old RedTg mice. FA, fatty acids; CHO, carbohydrates

Thus, RedTg mice had a modest increase in survival, exhibited physiological aspects of CR in the absence of reduced energy intake, and, importantly, were protected against DEN‐induced liver carcinogenesis. Based on our results summarized in Figure 6, we propose that these beneficial effects were conferred through two interrelated processes: (i) protection against metabolic and oxidative stress, which leads to better maintenance of redox state and bioenergetics, and (ii) increased NADH oxidation, which promotes sirtuin activation and suppression of inflammation and cellular proliferation. In line with this, overexpression of SIRT1 confers protection against hepatic DNA damage and associated liver cancer caused by metabolic syndrome (Herranz et al., 2010). Further studies will be needed to unveil the molecular mechanisms of sirtuins in the remodeling of chromatin via epigenetic modification of histone and nonhistone proteins [reviewed in Ref. (Hwang, Yao, Caito, Sundar & Rahman, 2013; Xie, Zhang & Zhang, 2013)]. In conclusion, enhancement of metabolic flexibility coupled with efficient redox control and energy homeostasis underlies the positive influence of CYB5R3 and NQO1 co‐expression on health and longevity.

## 4 EXPERIMENTAL PROCEDURES

### 4.1 Transgenesis, animal models, and diets

NQO1‐transgenic mice overexpressing the rat *Nqo1* gene (Figure S4) and CYB5R3‐Tg mice that overexpress the rat *Cyb5r3* gene (Martin‐Montalvo et al., 2016) were employed to generate RedTg mice. Group‐housed mice were maintained in a room at constant temperature (20°–22°C) with 30%–70% relative humidity and 12‐h light:dark cycles. Mice were fed ad libitum on a standard diet [*SD* ‐ *(*#110700—Research Diets, Bethlehem, PA)]. For the longevity study, male RedTg (*n* = 75) and wild‐type (Wt) (*n* = 64) mice were employed and their body weight was monitored monthly. The longevity study was performed in parallel with a previous study (Martin‐Montalvo et al., 2016), and therefore, the same values for the Wt group of mice are represented in both studies. For the diethylnitrosamine (DEN)‐induced liver cancer study, male RedTg (*n* = 18) and Wt (*n* = 17) mice were fed a high‐fat AIN‐93G Purified Rodent Diet [HF diet ‐ (#101920 ‐ Research Diets)] at the indicated time of the experiment, and their body weight was measured biweekly. The composition of SD and HF diets can be found in Tables S1 and S2. All animal protocols were approved by the ACUC of the National Institute on Aging, an AAALAC‐accredited institution (protocol #277‐TGB‐2019).

Animals were inspected daily for health issues, and deaths were recorded for each animal. The criteria for euthanasia of moribund animals were based on an independent assessment by a veterinarian, according to AAALAC guidelines, and only cases where the condition of the animal was considered incompatible with continued survival were represented as deaths in the curves. Animals removed at sacrifice for experimental procedures were treated as censored deaths. Survival curves were plotted using the Kaplan–Meier method, which includes all animals at each time point.

### 4.2 Fasting glucose and insulin determination

A 6‐h fasted protocol was used for the determination of blood glucose and plasma insulin levels in mice as described in the Supporting Information.

### 4.3 Oral glucose and insulin tolerance tests (OGTT and ITT)

OGTT and ITT were performed as previously described (Martin‐Montalvo et al., 2013). At the time points indicated, glucose was measured in whole blood by tail venipuncture using the Ascensia Elite glucose meter.

### 4.4 Homeostasis model assessment of insulin resistance (HOMA‐IR)

Insulin resistance was estimated using the HOMA2 Calculator software available from the Oxford Centre for Diabetes, Endocrinology, and Metabolism Diabetes Trials Unit website as described (Levy, Matthews & Hermans, 1998).

### 4.5 Body composition

A minispec LF90 nuclear magnetic resonance (NMR) spectrometer (Bruker Optics, Billerica, MA) was employed to acquire measurements of lean, fat, and fluid masses in live mice.

### 4.6 Metabolic clamps

At the age of 14 months, mouse metabolic rate during a fed‐fast–refed cycle was assessed by indirect calorimetry in open‐circuit Oxymax chambers (CLAMS; Columbus Instruments, Columbus, OH), as previously described (Martin‐Montalvo et al., 2016). Movement (both horizontal and vertical) was monitored with light beams, yielding counts of beam breaks for each mouse. All mice were acclimatized to the monitoring cages for 4 hr prior to recording.

### 4.7 Physical performance tests

Various physical performance tests were performed on 14‐month‐old RedTg and Wt mice according to standard procedures as described in the Supporting Information.

### 4.8 Gel electrophoresis and western blotting

Preparation of mouse liver and skeletal muscle homogenates from 14‐month‐old RedTg and Wt mice was followed by protein separation by gel electrophoresis and detection of specific proteins according to standard procedures as described in the Supporting Information. Antibodies used are presented in Table S3.

### 4.9 Measurements of mitochondrial activities

Activities of NADH:coenzyme Q oxidoreductase (complex I), succinate dehydrogenase (complex II), ubiquinol:cytochrome c oxidoreductase (complex III), cytochrome c oxidase (complex IV), and citrate synthase (CS) were determined in mouse livers by spectrophotometric assays as described (Spinazzi, Casarin, Pertegato, Salviati & Angelini, 2012).

### 4.10 Untargeted metabolomics

Metabolomics analysis on liver extracts from 14‐month‐old RedTg and Wt mice was performed at the UC Davis West Coast Metabolomics Center as previously described (Mitchell et al., 2016). Normalization was performed as described in the Supporting Information.

### 4.11 Targeted metabolomics

Sample preparation and LCMS‐based quantitative measurement of NAD^+^ and NADP^+^ in muscle extracts from 14‐month‐old RedTg and Wt mice was performed in the Department of Biochemistry, University of Iowa, as previously described (Trammell & Brenner, 2013).

### 4.12 Gene expression

Total RNA was extracted from frozen liver tissue using TRIzol^®^ reagent (Thermo Fisher Scientific, Waltham, MA). Total RNA concentration and quality were measured by Nanodrop (Thermo Fisher, Waltham, MA, USA) and by Agilent Bioanalyzer RNA 6000 chip (Agilent, Santa Clara, CA). cDNA reverse transcription and quantitative RT–PCR were carried out according to standard procedures as described in the Supporting Information. The primer sequences are presented in Table S4. Full methodological details including microarray analysis are described in the Supporting Information.

### 4.13 Diethylnitrosamine (DEN)‐induced hepatocellular carcinoma model

Sixteen‐day‐old mice were injected intraperitoneally with DEN at a dose of 5 mg/kg body weight, and, at 21 days of age, they were switched to a HF diet for 16 months as previously described (Martin‐Montalvo et al., 2016). Magnetic resonance imaging (MRI) was performed on 9‐month‐old mice to determine liver and tumor volumes as described below. At the conclusion of the study, the surviving mice were euthanized.

### 4.14 Magnetic resonance imaging

Mice were anesthetized using 1%–2% isoflurane in oxygen at a flow rate of 1.5 lpm and placed in a Bruker Biospec 7T/30 cm MRI scanner (Bruker Biospin, Ettlingen, Germany) equipped with a 72‐mm resonator coil (Bruker). Full methodological details for the detection and quantification of liver tumors are described in the Supporting Information.

### 4.15 Statistical analysis

Unless otherwise stated, statistical comparisons between genotypes were performed by two‐tailed Student's *t* test using Excel 2010 (Microsoft Corp., Redmond, WA, USA). For survival studies, a log‐rank statistical test was used. Analyses were performed using GraphPad Prism v. 6 (GraphPad Software, Inc., La Jolla, CA). In all experiments, results are represented as the mean ± *SEM*, with *p *≤* *.05 considered significant.

## ADDITIONAL INFORMATION

*Accession codes*: Microarray data have been deposited in the Gene Expression Omnibus database under accession code GSE108379.

## CONFLICT OF INTEREST

The authors declare no conflict of interest.

## AUTHORS’ CONTRIBUTION

K.J.P. A.D.R., and R.de C designed all experiments. K.J.P and A.M.M performed the longevity study. A.D.R., M.L., J.G., and F.F. carried out whole‐body physiology and physical performance studies. M.L. performed ELISAs. J.G. and H.M. performed MRI data analysis [tumor burden in liver of DEN‐treated mice]. M.A.A. performed metabolomics analysis. A.D.R, M.L, J.G., M.C.R., and A.S.P. carried out Western blot, microarray, and qPCR analyses. K. W. F. performed MRI scanning. A.D.R., A. di F., P.N., J.M.V, M.B., and R.d.C. contributed to the analysis and interpretation of the data, and the production of the manuscript. All authors critically reviewed and approved the manuscript.
