# Supplementary material for: Overexpression of CYB5R3 and NQO1, two NAD +‐producing enzymes, mimics aspects of caloric restriction
Source: Aging Cell. 2018 Apr 28;17(4):e12767. doi: 10.1111/acel.12767 (PMC6052403; doi:10.1111/acel.12767)
Supplement: Supplementary file 1 [file ACEL-17-na-s001.pdf]

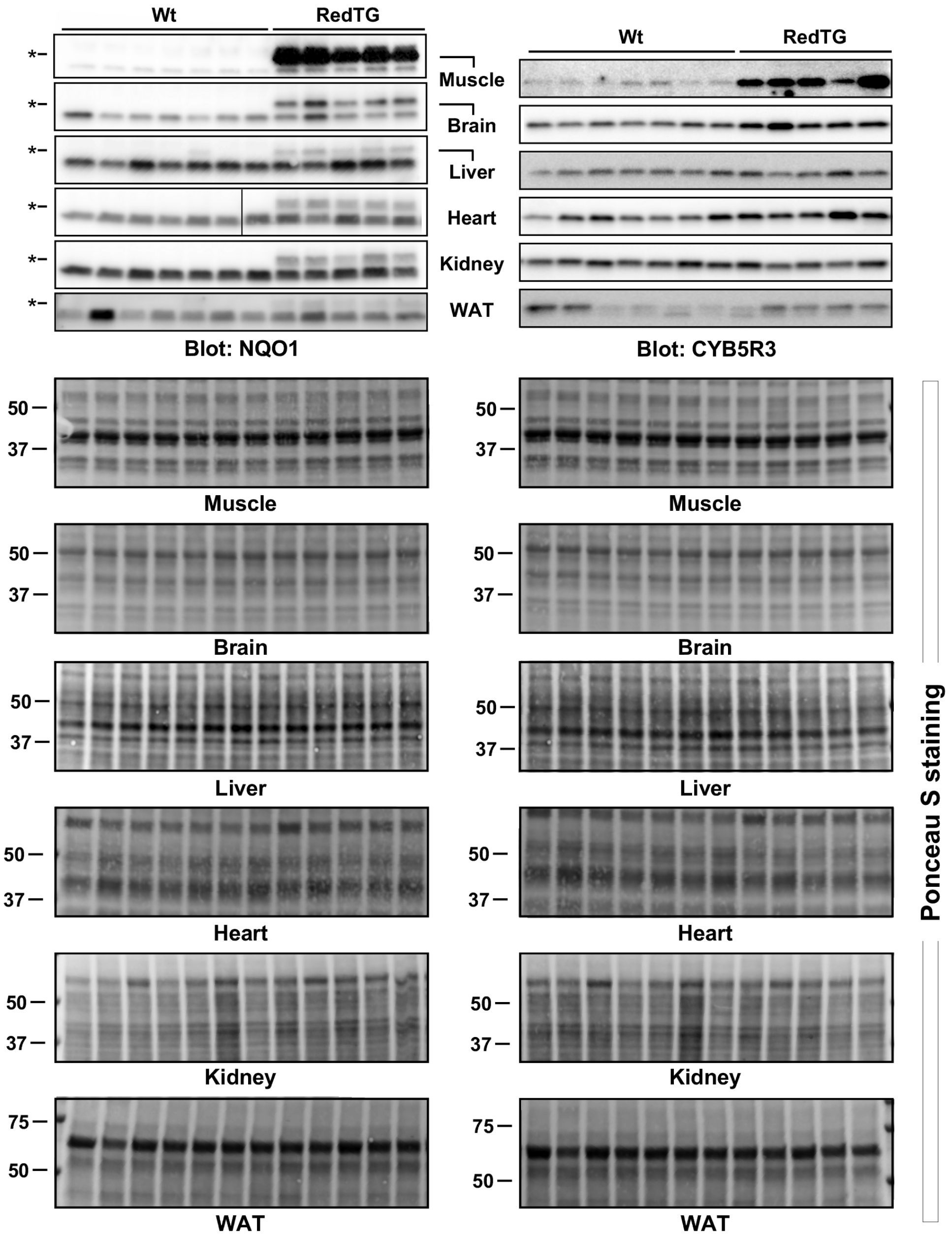

Fig. S1. Validation of CYB5R3 and NQO1 overexpression in RedTg mice. Immunoblots of NQO1 (left panels) and CYB5R3 (right panels) in the following mouse tissues: Muscle, brain, liver, heart, kidney, and white adipose tissue (WAT) from WT (n=7) and RedTg (n=5) mice. Ponceau S staining of the nitrocellulose membranes confirmed comparable loading in each lane. *The rat NQO1 transgene protein product migrates slower than the mouse homolog while the rat CYB5R3 transgene protein product have similar migration. Please note that NQO1 is not overexpressed in WAT and CYB5R3 in kidney and liver.

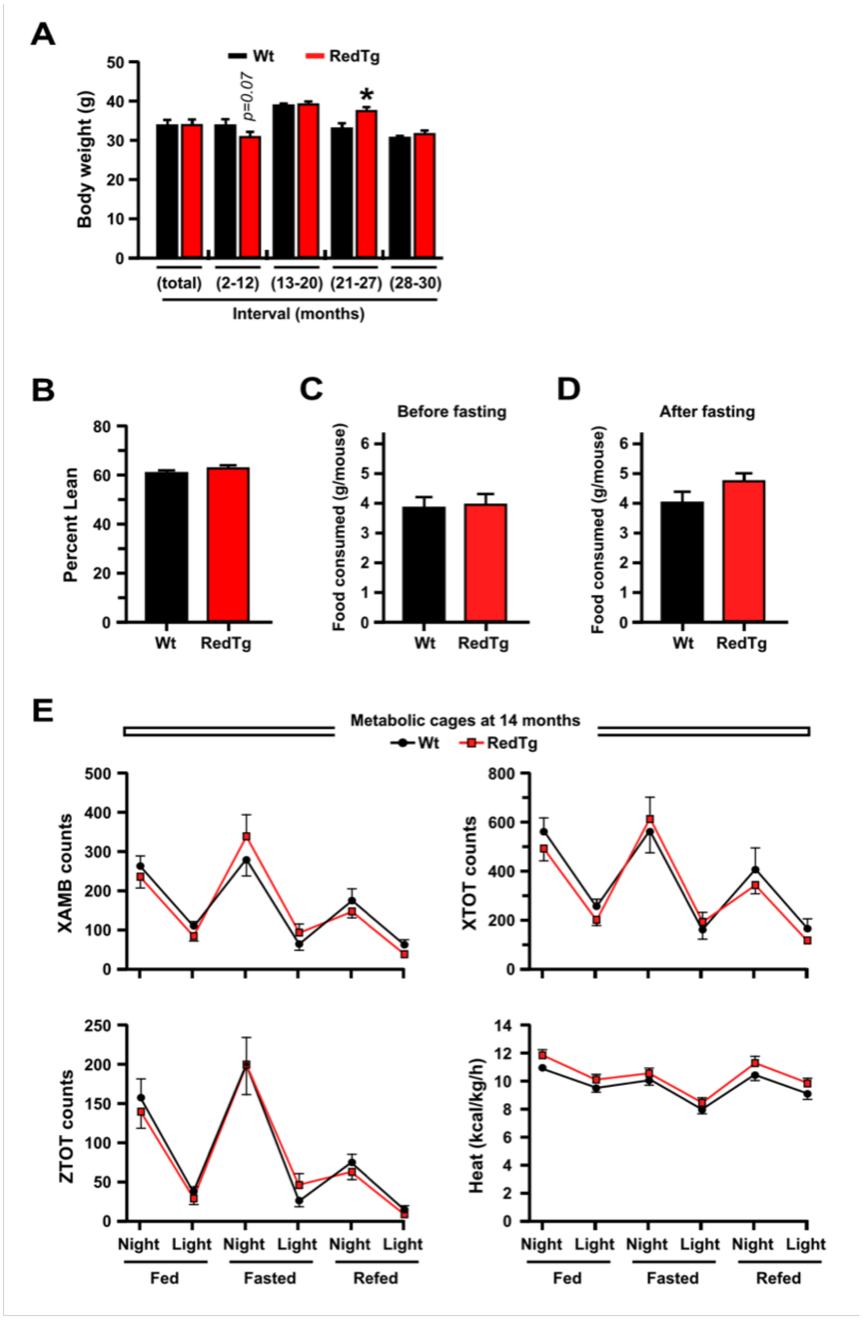

Fig. S2. Metabolic and behavioral responses in RedTg mice. (A) Averaged total body weight throughout the lifespan (2-30 months) and at the indicated intervals (in months). (B) Lean percentage was determined by nuclear magnetic resonance (NMR) in 14 months-old mice, n= 5-8 mice per group. (C-E) Fourteen months-old mice were placed into metabolic cages and subjected to a fed-fasted-refed cycle that lasted 72 h. Food consumption was calculated before and after the fasting period. Spontaneous activity and heat production by the mice held in the metabolic chambers, n=6-8 mice per group. Data are represented as the mean ± SEM. *$p < 0.05$ (B-E) No significant differences were found.

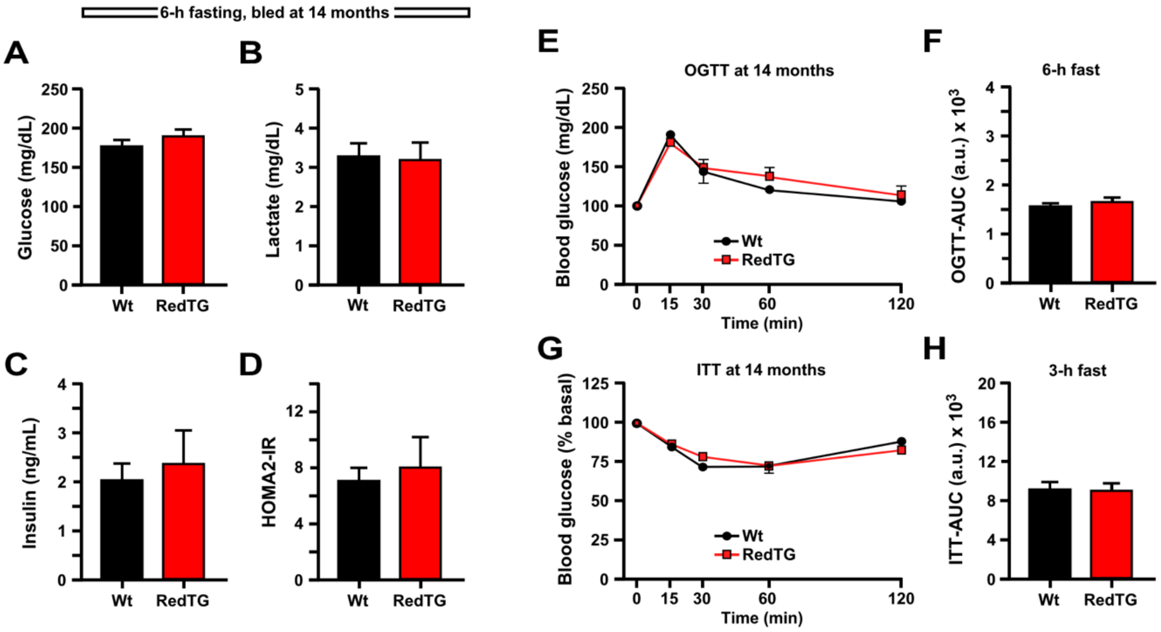

Fig. S3. Peripheral glucose homeostasis in RedTg mice. (A, B) Blood glucose and lactate levels and (C) serum insulin levels were determined after a 6-h fast, n=5-9 mice per group. (D) HOMA2-IR index, n=5-7 per group. (E) Blood glucose levels were measured over a period of 2 h during an OGTT, n=5-7 per group. (F) OGTT-AUC was calculated. (G) Blood glucose levels were measured over a period of 2 h during an ITT, n=5-8 per group. (H) ITT-AUC was calculated. Data are represented as the mean ± SEM. No significant differences were found.

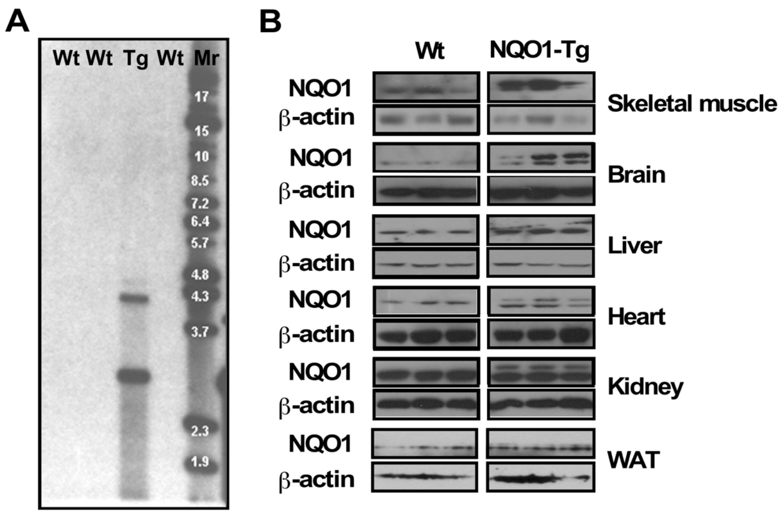

Fig. S4. Generation of NQO1-Tg mice. (a) Representative southern blotting analysis of NQO1-Tg mice. Genomic DNA from NQO1-Tg and Wt mice were digested with EcoRV, blotted and hybridized with a labeled DNA probe. DNA markers are shown on the right (Mr). (c) Western blots showing the levels of NQO1 in several tissues. WAT; white adipose tissue; BAT; brown adipose tissue. n = 3 per group.

Table S1, Ingredients of Standard Diet (SD)-AIN-93G

Ingredient	$g.kg^{-1}$	$Kcal.kg^{-1}$
Casein, High Nitrogen	200	716
L-cysteine	3	12
Sucrose	100	400
Cornstarch	397.1	1429.5
Dextrose	132	501.6
Soybean Oil	70	630
t-Butyl hydroquinone	0.014	0
Cellulose	50	0
Mineral Mix#210025	35	30.8
Vitamin Mix#310025	10	38.7
Choline Bitartrate	2.5	0

AIN-93G purified rodent diet (DYET#110700, Revised). Ingredients for food composition are shown as $g.kg^{-1}$ and $kcal.kg^{-1}$.

Table S2, Ingredients of High Fat (HF) Diet-AIN-93G

Ingredient	$g.kg^{-1}$	$Kcal.kg^{-1}$
Casein, High Nitrogen	213	762.54
Soy protein Isolate	103	379.04
Maltrodextrin	62.1	235.98
Cornstarch	397.1	1429.5
Soybean Oil	43.009	387
Hydrogenated Coconut Oil	294	2646
DL-Methionine	3	12
L-cysteine	3	12
Cellulose	66.141	0
Mineral Mix#213036	35	86.46
Vitamin Mix#310025	16.61	64.2807
Calcium Phosphate, dibasic	25.14	0
Calcium Carbonate	4.9	0
Choline Bitartrate	2.5	0

AIN-93G purified rodent diet (DYET#101920, Revised). Ingredients for food composition are shown as $g.kg^{-1}$ and $kcal.kg^{-1}$.

Table S3, List of commercial antibodies

Symbol	Company	#Catalog	Dilution
NQO1	Abcam	ab2346	1/2000
CYB5R3	Proteintech	10894-1-AP	1/10000
HKII	Cell Signaling	2867S	1/1000
PFK	Cell Signaling	8175	1/1000
LDHA	Santa Cruz	SC-27230	1/500
PDH	Cell Signaling	2784S	1/500
PGC1α	Abcam	ab54481	1/1000
SIRT3	Abcam	ab86671	1/500
ACAA2	Abcam	ab140529	1/40000
HADHSC	Santa Cruz	SC-74650	1/40000
GLUT-1	Santa Cruz	SC-7903	1/1000
GCK	Santa Cruz	sc-17819	1/1000
SIRT1	Sigma	S5196	1/1000
Acetyl-Lysine	Immunechem	ICP0380	1/2000
pACC	Millipore	07-303	1/1000
β-actin	Abcam	Ab8227	1/1000

Table S4. Primer pair sequences used for quantitative RT-PCR analysis

Gene	Forward primer sequence (5'–3')	Reverse primer sequence (5'–3')
**MsIL1β**	CCAAGCAATACCCAAAGAAGAA	TTAGAAACAGTCCAGCCCATAC
**MsTnfrs18**	GGGAGCAGACAGAAGAAAAGTG	TCGTAACTCACCGCTCTCATAC
**MsGpx1**	CCTGACATAGAAACCCTGCTGT	TTCATTAGGTGGAAAGGCATC
**MsGadd45b**	AAGAGAGCAGAGGCAATAACCA	GAAGGTATCACGGGTAGGGTAG
**MsTsc22d1**	ATCAGAGAGTGAGCAGGGATGT	TTTGAAGGGAAGGAGAAGAGAG
**MsGAPDH**	CACCAACTGCTTAGCCCC	TCTTCTGGGTGGCAGTGATG

Ms: Mouse.

Table S5. Analysis of muscular hepatic mitochondrial activity of RedTg vs. Wt mice.

Muscle	Symbol	Wt	RedTg	P value
Complex I	% of activity / unit of CS	16.08 ± 0.83	15.10 ± 1.26	0.78
Complex II	% of activity / unit of CS	23.34 ± 1.92	37.67 ± 3.85	0.15
Complex III	% of activity / unit of CS	108.68 ± 6.39	122.05 ± 9.89	0.63
Complex IV	% of activity / unit of CS	89.95 ± 7.79	96.20 ± 7.83	0.83
CS	Specific activity (µmol/min/mg prot)	290.11 ± 18.06	276.42 ± 17.50	0.83

Liver	Symbol	Wt	RedTg	P value
Complex I	% of activity / unit of CS	43.90 ± 3.60	42.22 ± 3.55	0.75
Complex II	% of activity / unit of CS	58.41 ± 10.99	71.44 ± 13.28	0.46
Complex III	% of activity / unit of CS	19.35 ± 4.52	19.64 ± 2.76	0.96
Complex IV	% of activity / unit of CS	102.31 ± 14.95	93.33 ± 12.66	0.67
CS	Specific activity (µmol/min/mg prot)	129.02 ± 4.19	128.78 ± 2.34	0.96

n=5-7 mice per group. Mitochondrial activities were expressed as percentage of CS activity in units. Data are represented as the mean ± SEM. CS: Citrate synthase.

Table S6. List of top 20 up-regulated transcripts in liver of RedTg vs. Wt mice.

PROBE_INFO	Symbol	zratio	fold change
NM_153558	Obp2a	5.87	5.27
NM_031368	Bglap3	3.04	2.59
NM_145067	Gucy2c	2.77	2.74
NM_009366	Tsc22d1	2.63	2.05
NM_080448	Srgap3	2.59	2.46
NM_001032298	Bglap2	2.58	2.29
XM_006501516	Prok1	2.56	2.28
NM_177406	Cyp4a12a	2.53	1.89
NM_139051	Nr5a1	2.43	2.32
NM_001111288	Sco2	2.28	1.84
NM_138302	Tymp	2.22	1.85
NM_001104531	Cyp2d11	2.18	1.53
NR_033567	E130304I02Rik	2.13	1.8
NM_013651	Sf3a2	2.13	1.67
NR_028266	BC037032	2.05	1.78
NM_024272	Ssbp2	2.05	1.81
NM_153778	Atoh8	2.02	1.9
NM_001162944	Gm10318	1.99	1.72
NM_026159	Retsat	1.99	1.6
NM_001168502	Zfp57	1.98	1.86

Table S7, List of top 20 down-regulated transcripts in liver of RedTG vs. Wt mice.

PROBEINFO	Symbol	zratio	fold change
NM_009791	Aspm	-3.9	-3.56
NM_146187	Ffar2	-3.9	-4.13
NM_021718	Ms4a4b	-3.9	-4.89
NM_010090	Dusp2	-3.97	-4.88
NM_010389	H2-Ob	-4.01	-4.37
NM_008518	Ltb	-4.15	-5.99
NM_001033186	Skap1	-4.17	-4.62
NM_020044	Lat2	-4.2	-5.19
NM_145141	Fcrla	-4.21	-5.2
NM_009400	Tnfrsf18	-4.28	-5.61
NM_009850	Cd3g	-4.29	-4.74
NM_015811	Rgs1	-4.41	-4.6
NM_172435	P2ry10	-4.54	-4.9
BC019425	Igh-VJ558	-4.56	-4.78
NM_025427	Rgcc	-4.68	-5.18
NM_020034	Hist1h1b	-5.27	-6.8
NM_009835	Ccr6	-5.31	-9.57
NM_152839	Jchain	-5.37	-9.59
NM_011136	Pou2af1	-5.67	-10.15
NM_027222	Mzb1	-5.7	-12.77
